# Photosensitivity Reaction From Operating Room Lights After Oral Administration of 5-Aminolevulinic Acid for Fluorescence-Guided Resection of a Malignant Glioma

**DOI:** 10.7759/cureus.13442

**Published:** 2021-02-19

**Authors:** Alexander T Yahanda, Gavin P Dunn, Michael R Chicoine

**Affiliations:** 1 Department of Neurosurgery, Washington University School of Medicine, St. Louis, USA

**Keywords:** 5-aminolevulinic acid, photosensitivity, glioma resection, tumor fluorescence, operating room

## Abstract

Orally administered 5-aminolevulinic acid (5-ALA), which was approved in the United States in 2017, is preferentially metabolized by malignant glioma cells into protoporphyrin IX and enhances tumor visualization when using a blue light filter on an operating microscope. Photosensitivity after 5-ALA administration is a known side effect, but a photosensitivity reaction from operating room lights has not yet been documented. We report the case of a 56-year-old man with a history of previous resection of a grade II astrocytoma who presented with imaging concerning for tumor recurrence and possible malignant transformation. Repeat surgical resection utilized 5-ALA. Soon after the surgery, he developed reddening of his skin, particularly over the right side of his head and neck, with blistering and peeling in a distribution that was particularly exposed to operating room lights during surgery. No other areas of his skin experienced the same redness, blistering, or peeling. Topical lotions were applied and the skin changes resolved spontaneously over weeks. Significant photosensitivity after administration of oral 5-ALA is a rare complication, but neurosurgeons who perform fluorescence-guided tumor resection should remain cognizant of its potential association with exposure to intense light, including in the operating room. Phototoxicity typically is self-limited, but awareness is important to minimize its occurrence.

## Introduction

Five-aminolevulinic acid (5-ALA) is an orally administered intraoperative optical imaging agent that was approved by the United States Food and Drug Administration in June 2017 for patients with suspected World Health Organization (WHO) grade III or IV gliomas [1]. It has been approved in Europe since 2007. Biochemically, 5-ALA is a precursor of protoporphyrin IX in the heme biosynthesis pathway. Its utility as a tumor-specific fluorescent agent is based on the idea that malignant gliomas selectively uptake 5-ALA via the disrupted blood-brain barrier and metabolize it to protoporphyrin IX. Once produced and retained in glioma cells, protoporphyrin IX fluoresces red/pink when exposed to 370-440 nm wavelength blue light, and this fluorescence can be viewed through a specialized filter [2]. Both eliciting and viewing tumor fluorescence can be accomplished with specially equipped neurosurgical microscopes [2].

Previous investigations, including randomized prospective trials, have shown that 5-ALA can increase the extent of resected tumor and progression-free survival compared to traditional microsurgery without increase in neurological deficits [1,3]. Consequently, 5-ALA is a widely used surgical adjunct in Europe and Asia and has become increasingly more common in the United States since its approval. Side effects of 5-ALA are very rare [4,5], but photosensitization has been associated with its use, typically within 24 hours after administration [6]. However, photosensitivity reactions after oral 5-ALA administration have not yet been documented as occurring secondary to operating room lights. Here, we report the case of a patient who developed such a photosensitivity reaction after oral 5-ALA administration for resection of a malignant glioma.

## Case presentation

**Figure 1:**
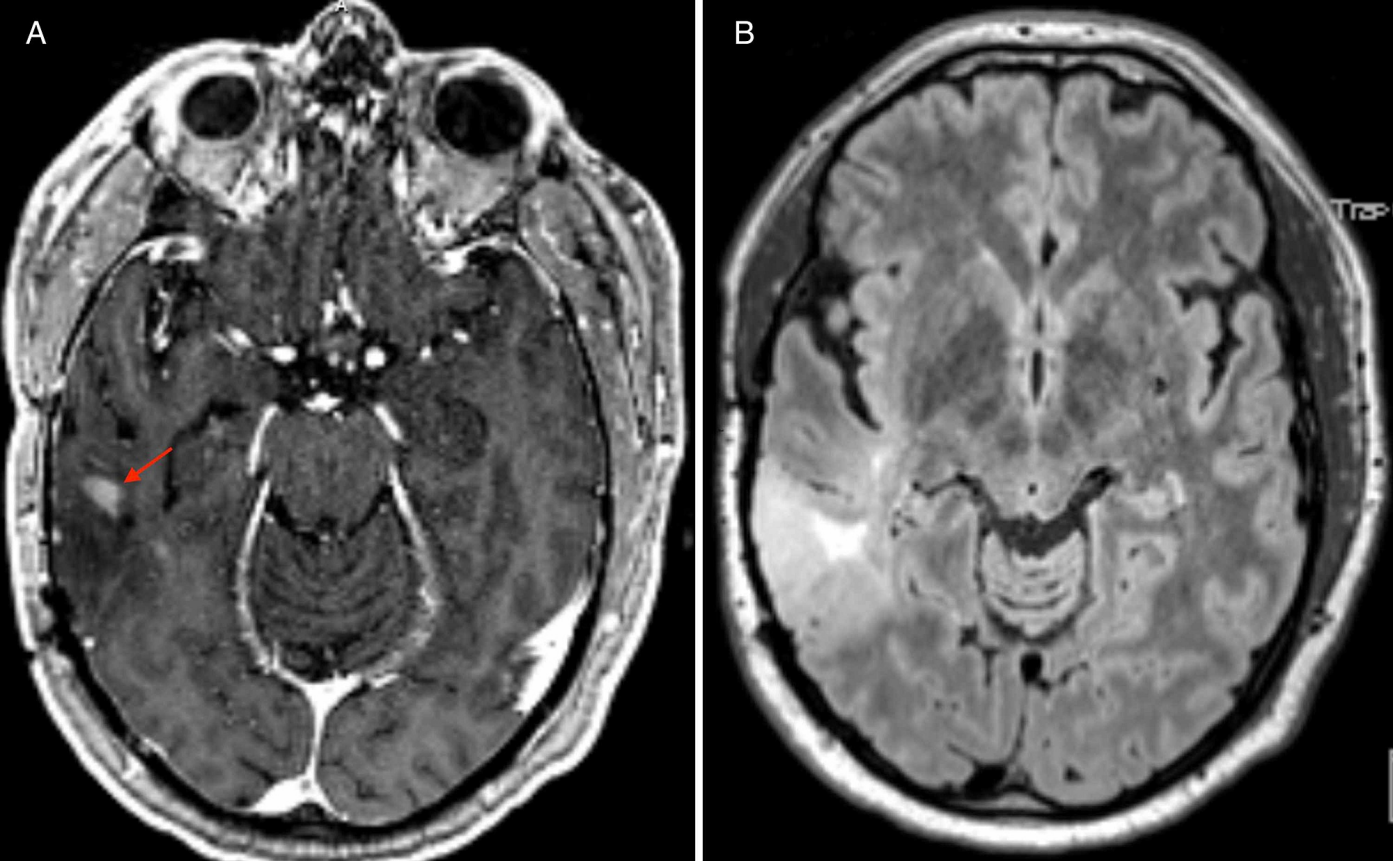
Preoperative T1-weighted MRI with contrast demonstrating an enhancing focus (red arrow) in the right posterior temporal lobe adjacent to the resection cavity for the patient’s prior grade II astrocytoma (A). Preoperative FLAIR MRI demonstrating hyperintensity in the right posterior temporal lobe, which had increased in size from previous studies (B). Pathology obtained during surgery confirmed WHO grade III anaplastic astrocytoma. MRI, magnetic resonance imaging; FLAIR, fluid-attenuated inversion recovery

A 56-year-old man with a history of a WHO grade II astrocytoma for which a right posterior temporal lobe resection (without 5-ALA as it had not yet been approved in the United States) had been performed 15 years previously at another institution presented with an episode of left arm stiffening, aphasia, and altered mental status that was concerning for a new-onset seizure. Magnetic resonance imaging (MRI) studies demonstrated an enlarging mass with enhancing foci adjacent to the previous resection cavity, likely representing tumor recurrence and malignant transformation (Figure 1). He was referred to our institution for further management, and surgical resection was recommended.

**Figure 2:**
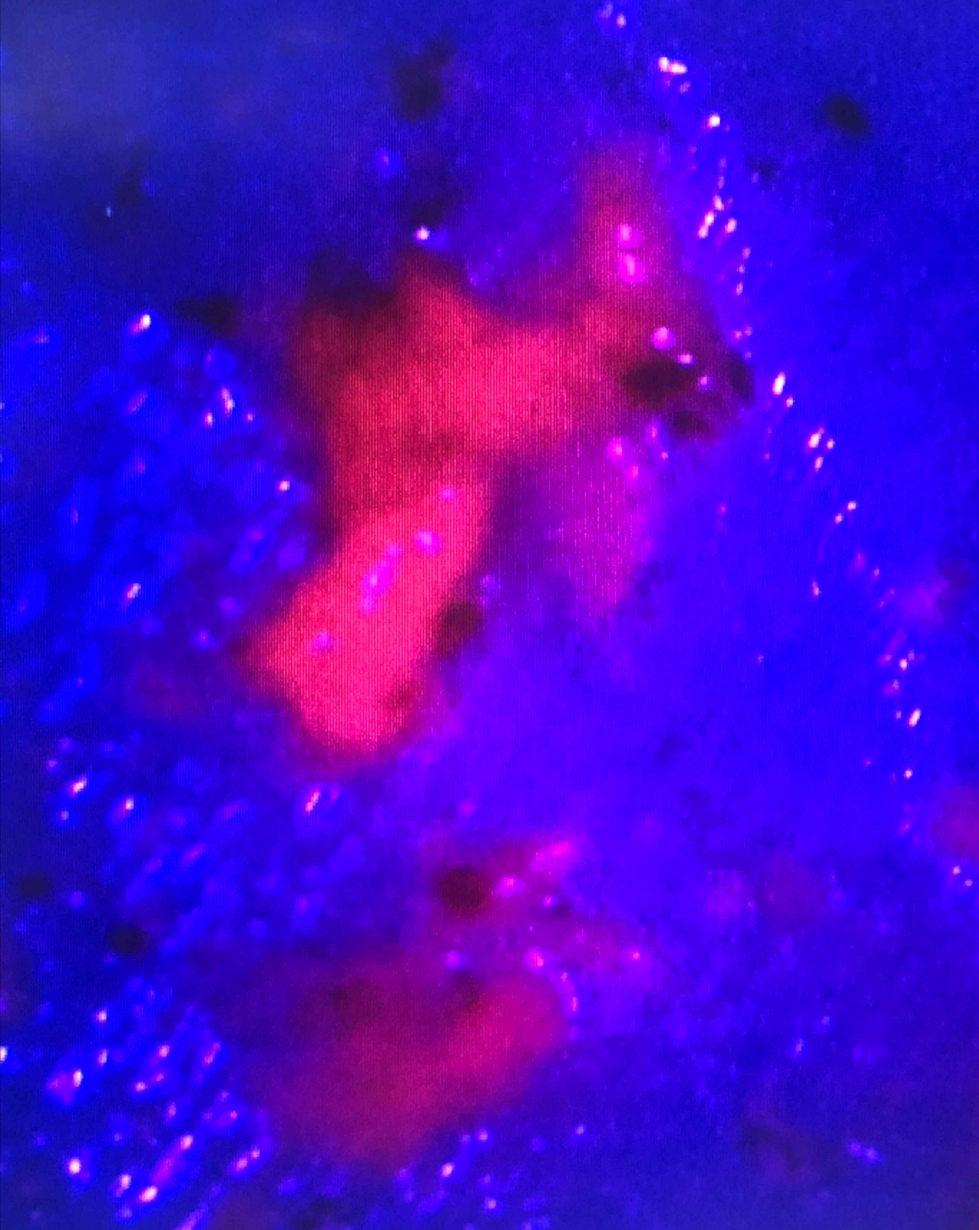
Tumor that was resected with the aid of 5-ALA (pink color). The tumor demonstrates red/pink fluorescence when the appropriate blue light and filter are used with the operating microscope. 5-ALA, 5-aminolevulinic acid

The patient underwent a repeat right temporoparietal craniotomy. In accordance with the hospital protocol, he was given oral 5-ALA (GleolanTM NX Development Corporation, Lexington, KY, USA) at the standard dose of 20 mg/kg three hours preoperatively, which successfully yielded tumor fluorescence when viewed with the specialized blue filter on the operative microscope (Zeiss Pentero microscope, Carl Zeiss Meditec, Inc., Dublin, CA, USA) (Figure 2). The tumor was resected until the surgeon felt that maximal resection had been achieved. To confirm this extent of resection, intraoperative MRI (iMRI) studies were obtained using a 1.5 Tesla movable iMRI device (IMRIS, Inc., Minnetonka, MN, USA). These images demonstrated resection of all enhancements with minimal residual T2/fluid-attenuated inversion recovery abnormality in the posterior aspect of the resection cavity, which was subsequently resected prior to closure. The patient tolerated surgery well with no new neurological deficits and was discharged on postoperative day two. Surgical pathology confirmed a WHO grade III anaplastic astrocytoma with an isocitrate dehydrogenase 1 R132H point mutation, and the patient was referred for chemotherapy and radiation therapy.

**Figure 3:**
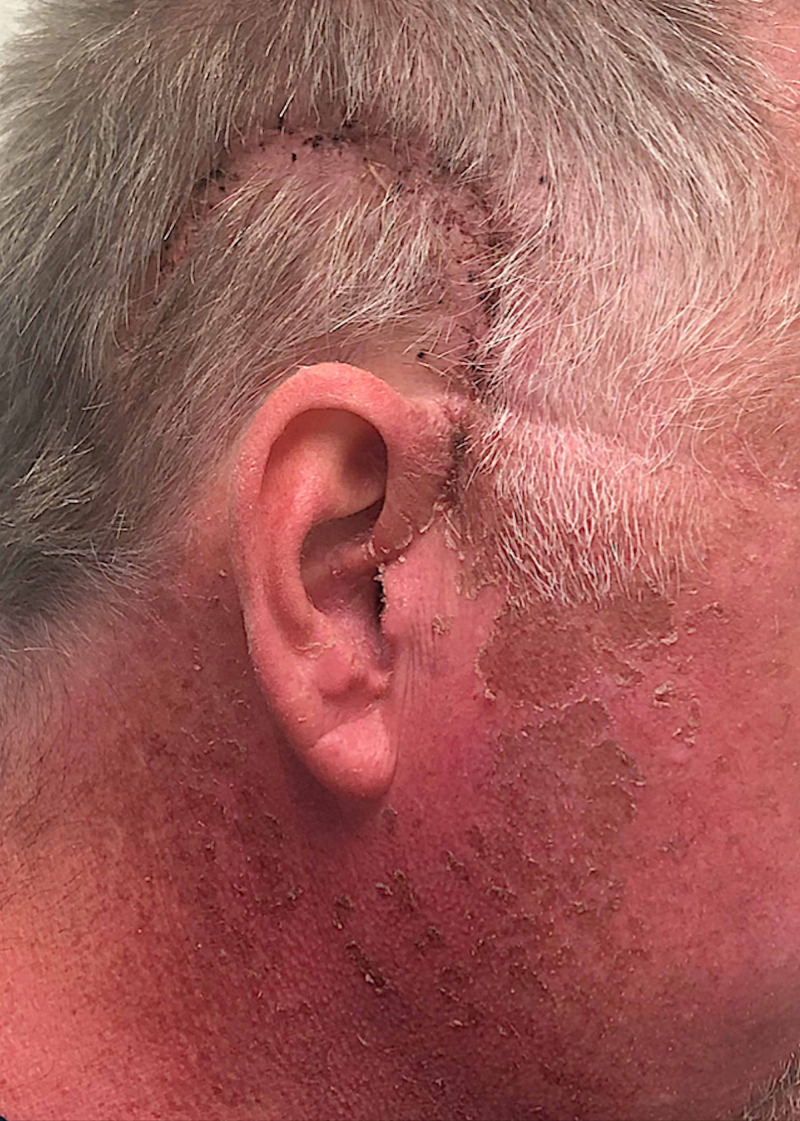
Image of the right side of the patient’s head and neck taken at his one-week follow-up visit demonstrating a healing surgical incision as well as skin erythema, blistering, and peeling. Of note, the worst of the skin erythema and the blistering and peeling were only seen on the right side of his head/neck, which were the areas of his body that had been exposed to surgical lights in the operating room.

During his hospitalization, in the early postoperative period, the patient developed mild diffuse erythema of his skin, including on his extremities and torso. The intensity of the erythema was most severe on the right side of his head and neck where there was associated blistering and peeling of the skin in a distribution defined by the region of the surgical field (Figure 3). Notably, the right side of his head and neck were the only areas of his body that had been exposed to the intense surgical lights (Berchtold Chromophare halogen lights, Stryker, Kalamazoo, MI, USA) in the operating room. Similar erythema and blistering was not observed on any other part of his body, including the left side of his head and neck, which had remained covered during surgery. He had not been exposed to any sources of natural light during his hospitalization and had been kept in a dark room postoperatively. No reddening of skin had been observed before surgery. He had no renal disease that may have compromised excretion of 5-ALA. The blistering on his head and neck persisted until his one-week postsurgical follow-up visit. He never experienced fever, rashes, or other symptoms during this time period. He treated the blisters with over-the-counter lotion and they resolved uneventfully over a few weeks. Consultation with dermatology was recommended to the patient, but he did not pursue it as the skin changes were resolving spontaneously.

## Discussion

Photosensitivity reactions are a rare but acknowledged side effect of oral 5-ALA administration, and patients are generally advised to avoid direct light for 24-48 hours after the agent is administered. The link between 5-ALA and tissue photosensitization has been well-established, as topical or oral 5-ALA has been used in multiple fields to enhance photodynamic therapy for treatment of lesions of the skin, oral mucosa, and esophagus [7-9]. Here, we report an instance in which a patient developed a photosensitivity reaction on areas of his body that had been exposed to operating room lights. To the authors’ knowledge, this is the first such reported case related to oral 5-ALA. This report should reinforce the need to warn patients and families about photosensitivity side effects that, while not fatal or permanent, could be distressing if unanticipated.

Multiple past reports have been published detailing photosensitivity reactions that resulted from operating room light exposure, but these concerned patients who had erythropoietic protoporphyria [10,11] or who were preoperatively administered methylene blue [12,13]. Erythropoietic protoporphyria is an X-linked disorder of porphyria metabolism that leads to the accumulation of protoporphyrin IX in cells of erythroid lineage [14]. When these cells are exposed to light at 320-595 nm wavelength, the high protoporphyrin IX levels lead to the generation of reactive oxygen species that mediate phototoxic effects on the skin [14,15]. Graham-Brown and Ilchyshyn [15] reported a case in which a patient with this disorder underwent surgery for the creation of a left arm arteriovenous fistula. The day after the surgery, the patient noted erythema and edema of the skin that had been exposed to operating room lights. No other areas of his body exhibited these findings. Herbert et al. [10] and McGuire et al. [11] detailed instances in which patients receiving liver transplants for erythropoietic protoporphyria experienced skin phototoxicity in areas that had remained uncovered during surgery.

Methylene blue is a dye that may be given intravenously during parathyroid gland surgery to identify abnormal glands. Upon exposure to light between 500-700 nm, the dye releases reactive oxygen species that can cause tissue damage [12]. Maguire et al. [12] presented two cases of patients who underwent parathyroidectomy using methylene blue. In both cases, the patient developed skin erythema and vesicles localized to the anterior neck and upper chest, areas that had been exposed during surgery. No erythema or other skin changes were noted in areas that had been covered. Lambrecht et al. [13] reported the case of a woman who developed a sharply demarcated photosensitivity reaction of the skin on her neck that was exposed to operating room lights. Of note, this case occurred right after the authors’ institution changed from halogen lamps to light-emitting diode (LED) lamps in the operating room. After emission spectrum analyses, these LED lights were found to produce more intense light in both the blue spectrum (430-490 nm) and the excitation spectrum for methylene blue (500-700 nm). The authors hypothesized that the combination of the photodynamic therapy-like effects of blue spectrum light and the photoexcitation of methylene blue contributed to skin phototoxicity.

Past reports examining photosensitivity after oral 5-ALA administration for glioma resection have found that this complication is quite rare, occurring in potentially <1% of patients [3,16]. The mechanism of 5-ALA-induced photosensitivity is similar to that of erythropoietic protoporphyrin. In addition to being taken up by malignant glioma cells, 5-ALA accumulates in skin cells, which convert it to protoporphyrin IX. The same light-induced generation of reactive oxygen species then leads to cytotoxic damage and the clinical signs and symptoms of photosensitization [17]. At the normal administered dose of 20 mg/kg body weight, 5-ALA reaches peak plasma levels at between 30 minutes and two hours, and return to baseline after 24 hours [16]. Protoporphyrin IX reaches maximum plasma levels in four hours, begins to decline over the next 20 hours, and is almost completely eliminated by 48 hours [18]. A pharmacokinetic study of 5-ALA found that an oral dose of 40 mg/kg body weight (twice the dose used for glioma resection) reached peak levels in the skin at a mean time of 6-10 hours, depending on the location, as measured by skin fluorescence [19]. After 40 hours, the average level of 5-ALA was 5% of the maximum.

Therefore, it is physiologically plausible that the patient in this report was rendered sufficiently photosensitized by oral 5-ALA to have a photosensitivity reaction to operating room lights. This hypothesis is further supported by the fact that only the skin that had been exposed to the lights in the operating room experienced the most severe erythema as well as blistering and peeling. Moreover, he did not have any other signs or symptoms to support a different medication-induced systemic reaction to 5-ALA (e.g., Stevens-Johnson syndrome). The skin peeling resolved with over-the-counter treatment, and the patient did not necessitate further medical treatment. Halogen lights, as used in this operation, produce more light towards the warm and infrared portions of the electromagnetic spectrum, and a wavelength of 635 nm is typically used for photodynamic therapy [8,9]. Perhaps this complication could have been avoided with diodes that emitted less light of these wavelengths, but this is difficult to know for sure. Specialized filters may be placed over operating room lights to eliminate phototoxic injury, though these would likely not be beneficial for the vast majority of cases that use 5-ALA.

It would be interesting to examine whether the standard 20 mg/kg dose of oral 5-ALA is necessary in all patients to achieve acceptable tumor fluorescence and maximal extent of resection or whether lower doses may yield satisfactory tumor detection while reducing the chances of photosensitivity reactions, as witnessed in this patient. Haj-Hosseini et al. [20] found that low-dose 5-ALA (5 mg/kg, administered orally) was equally reliable for tumor detection as the standard dosing, though the standard-dose cohort had higher intraoperative tumor fluorescence. Of note, no patients who were administered the lower dose were found to have protoporphyrin IX in their skin while it was detected in the skin of nearly every patient in the standard-dose cohort [20]. Further studies should examine the dosing of 5-ALA that is required for adequate tumor detection, as well as patient-specific factors that may affect the efficacy of these lower doses to minimize future incidences of photosensitivity. It is also not well-known if multiple doses of 5-ALA (e.g., across multiple tumor resections) increase the risk of photosensitivity reactions.

## Conclusions

Photosensitivity after administration of oral 5-ALA is a rare complication, and until now a report of 5-ALA-associated photosensitivity from operating room lights has never been published. Nonetheless, neurosurgeons who perform fluorescence-guided tumor resection should remain cognizant of this potential side effect. As 5-ALA becomes a more popular surgical adjunct for glioma resections, events like this will become more common. This report offers a practical teaching point for anyone involved in the care of patients who have undergone surgical resection utilizing this imaging agent. After administration of oral 5-ALA, the phototoxicity from exposure to light, particularly the intense lights of the operating room, may cause a significant skin reaction. Typically, this is a self-limited reaction, but minimizing light exposure is important to both reduce the chances of such a reaction as well as to avoid more serious skin damage or wound healing problems. Precautions may be taken such as shining the lights away from the patient until the patient has been fully draped or positioning the lights at the maximum distance from the patient that still offers adequate illumination.
